# A Cross-Sectional Study on the Awareness and Practice of the Use of Supplemental Vitamin C, Arginine, and Zinc in Managing Wounds Among Healthcare Workers in Saudi Arabia

**DOI:** 10.7759/cureus.51235

**Published:** 2023-12-28

**Authors:** Muazzam M Sheriff, Safaa D Alotaibi, Ghada H Alharbi, Mariam Mohammed O Alghamdi, Nawaf M Aljadani, Hend A Alzahrani, Shahenaz Hesham M Abdulhaq, Bushra A Mualla Aljuhani, Huda M Abdulaziz Sultan, Amira Esam A Ba Rahma, Rawabi D Alghamdi, Maram M Albishri, Nouf K Eldabai, Hassan A Mohammed Alharbi

**Affiliations:** 1 Microbiology and Immunology, Ibn Sina National College for Medical Studies, Jeddah, SAU; 2 Nutrition, King Fahad General Hospital, Jeddah, SAU; 3 Nutrition, King Abdulaziz Medical City, Jeddah, SAU; 4 Preventive Medicine, King Fahad General Hospital, Al Baha, SAU; 5 Pediatrics, King Abdullah Medical Complex, Jeddah, SAU; 6 Gastrointestinal Oncology Clinical Nursing, National Guard Hospital, King Abdulaziz Medical City, Jeddah, SAU; 7 Medicine, Ibn Sina National College for Medical Studies, Jeddah, SAU; 8 Medicine, University of Jeddah, Jeddah, SAU

**Keywords:** cross-section study, kingdom of saudi arabia (ksa), supplements, wound, pressure ulcer, knowledge, awareness, zinc, arginine, vitamin c

## Abstract

Introduction

The intricate connection between nutrition and compromised wound healing exposes patients to heightened risks of pressure ulcers, infections, and delayed recovery from wounds or traumatic injuries. In-depth scientific investigations have shed light on the potential of specialized nutritional supplements, combined with regular wound care, to significantly boost the management of pressure ulcers and the wound healing process. The study focuses on supplemental Vitamin C, Arginine, and Zinc due to their established roles in wound healing, aiming to assess the awareness and practice of healthcare workers in Saudi Arabia regarding these essential nutrients for effective wound management. This cross-sectional study aimed to assess awareness and practice among healthcare workers in Saudi Arabia regarding the use of supplemental Vitamin C, Arginine, and Zinc in managing wounds.

Methods

This study adopts a cross-sectional research design to explore the dynamics to assess the awareness and practice among healthcare workers about the use of supplemental Vitamin C, Arginine, and Zinc in managing wounds in Saudi Arabia. The research methodology encompasses developing and validating a questionnaire, data collection, and subsequent analysis. Thorough statistical analyses, encompassing descriptive statistics, validated assessment scales, and inferential statistics, were conducted using SPSS and Microsoft Excel to explore intricacy prevalence and severity relationships with various factors, maintaining a statistical power of 80% at a cutoff value of 0.05.

Result

In a cross-sectional survey of 510 healthcare professionals, the socio-demographic analysis revealed a predominant hospital workplace (61.56%), with pharmacists representing 10.19%, and comprehensive tabulation of response rates and p-values, while the knowledge and awareness assessment demonstrated varied understanding and perceptions of wound care supplements, including frequent encounters with pressure ulcers or wounds (36.5%), diverse awareness levels for Vitamin C, Arginine, and Zinc, with the collaboration and communication dynamics among healthcare workers, detailed in tabulated response rates and p-values.

Conclusion

The findings reveal a diverse understanding landscape, with varying levels of awareness, perceived effectiveness, and confidence in applying these supplements.

## Introduction

The intricate connection between insufficient nutrition and compromised wound healing exposes patients to heightened risks of pressure injuries, infections, and delayed recovery from surgical or traumatic injuries [1]. In-depth scientific investigations have shed light on the potential of specialized nutritional supplements, combined with regular wound care, to boost the wound healing process [2] significantly. Essential nutrients for wound healing encompass not only adequate energy and protein to support healing processes but also conditionally essential amino acids like arginine and glutamine [3]. Insufficient nutrition poses a significant threat to wound healing, increasing the risks of pressure injuries, infections, and delayed recovery from surgical or traumatic injuries [4]. Specialized nutritional supplements, alongside regular wound care, have shown the potential to enhance the wound healing process [4]. Essential nutrients crucial for wound healing include amino acids like arginine and glutamine and minerals such as zinc, selenium, and iron [5]. Deficiencies in vitamins A, C, and D are linked to delays in wound healing [6].

In the Middle East, the prevalence of pressure ulcers, a major healthcare challenge, ranges from 7% to 44.4% [7]. Saudi Arabia faces a hospital-acquired pressure ulcer rate of 7.5% [7]. Effective wound management and pressure ulcer prevention are integral to contemporary healthcare [8]. This study focuses on healthcare workers in Saudi Arabia, aiming to assess their awareness and practice of the use of Vitamin C, Arginine, and Zinc supplements in managing wounds. In-depth scientific investigations have shed light on the potential of specialized nutritional supplements, combined with regular wound care, to significantly boost the management of pressure ulcers and the wound healing process [9].

Arginine, a crucial amino acid, plays a vital role in cell growth, collagen deposition, and immune function [10]. It serves as a precursor for nitric oxide, known for its impact on wound healing. Zinc is essential for enzymes involved in wound healing, tissue repair, growth, and immune support [11]. Vitamin C contributes to collagen synthesis, immune modulation, and antioxidant capabilities, all crucial for wound healing [12]. The study aims to bridge the knowledge gap among healthcare workers and enhance wound care practices in the unique healthcare landscape of Saudi Arabia. This cross-sectional study addresses the gap in knowledge by investigating the awareness and practice of healthcare workers in Saudi Arabia regarding the use of supplemental Vitamin C, Arginine, and Zinc in wound management, aiming to inform interventions and enhance healthcare practices.

This cross-sectional study aimed to evaluate the awareness and utilization practices of healthcare workers in Saudi Arabia concerning supplemental Vitamin C, Arginine, and Zinc in wound management, with the overarching objective of guiding interventions and enhancing healthcare practices in the region.

## Materials and methods

Research design

This study employs a cross-sectional research design to investigate the dynamics of assessing awareness and practice among healthcare workers regarding the use of supplemental vitamin C, arginine, and zinc in managing wounds in Saudi Arabia. The research methodology encompasses the development and validation of a questionnaire, data collection, and subsequent analysis [13].

Questionnaire development

A comprehensive questionnaire was developed to evaluate the awareness and practice among healthcare workers regarding the use of supplemental vitamin C, arginine, and zinc in managing wounds in Saudi Arabia. The questionnaire comprises sections covering demographic details, professional experience, awareness and practice-based questions, psychosocial aspects, frequency of cases, hesitancy of application, training programs, skills, and validated assessment scales of patients' health [14].

Translation and cultural adaptation

To ensure cultural relevance and linguistic accuracy, a meticulous translation and adaptation process was undertaken for the questionnaire. Bilingual experts translated the English version into Arabic, followed by a separate back-translation by a different set of bilingual experts. Consensus resolution addressed any discrepancies, maintaining the original intent while aligning with the local cultural context [15].

Questionnaire validation

The translated questionnaire underwent validation to ensure reliability and validity. Content validation involved a panel of nutrition and statistician experts. A pilot study on a small sample size of 51 participants assessed the clarity and comprehensibility of questionnaire items, with participant feedback used to refine it [16].

Sample size

The study's sample size was determined using the G-power online sample calculator, aiming for a 95% confidence level with a margin of error within ±5%. The calculated sample size required was 510, based on inclusion criteria set by a specialist nutrition healthcare professional [17].

Data collection

Informed consent was obtained, emphasizing confidentiality and voluntary participation. The study took place from August 15, 2023 to November 13, 2023, with 510 participating volunteers. The primary objective was to assess the proportion of all categories.

Data analysis

A thorough statistical analysis was conducted on the collected data. Descriptive statistics presented demographic and intricacy-related variables [18]. Validated assessment scales were analyzed to quantify intricacy prevalence and severity [19]. Inferential statistics, including chi-square tests and regression analysis, explored relationships between intricacy and various factors. SPSS (IBM Corp., Armonk, NY) and Microsoft Excel were employed for data analysis, maintaining a statistical power of 80% at a cut-off value of 0.05 [20].

## Results

This comprehensive cross-sectional survey study gathered valuable responses from 510 participants, providing insights into the socio-demographic composition, awareness, and practice of healthcare workers towards the use of supplemental vitamin C, arginine, and zinc in managing pressure ulcers and wounds in Saudi Arabia. The study also procured responses providing insights on the information and training, barriers, and challenges regarding the use of supplemental vitamin C, arginine, and zinc in managing wounds in Saudi Arabia. The study also measured responses through a rating scale of 1 to 10 about the importance of nutritional supplements, including vitamin C, arginine, and zinc, in the management of wounds.

Socio-demographic characteristics

The majority of respondents were from the Western region (71.8%), followed by the Central (7.1%), Eastern (11.8%), Southern (7.1%), and Northern (2.4%) regions. Gender distribution revealed a predominance of female respondents (91.8%). The age distribution showed a significant percentage of respondents in the 25-34 years category (49.8%). In terms of professions, dietitians constituted the largest group (46.27%), followed by physicians (22.35%), nurses (16.47%), pharmacists (10.19%), and other healthcare professionals (4.70%). The distribution of experience varied, with the majority having less than 10 years of experience, and hospitals were the predominant workplace (61.56%). The survey questionnaire response rate absolute number (total=510) along with response rate percentage with p-value (p ≤ 0.05) for the socio-demographic characteristics were tabulated in detail (Table 1).

**Table 1:** Socio-demographic data

Survey Questionnaires	Response rate Absolute number (total=510)	Response rate Percentage	P-value (p ≤ 0.05)
Region			0.061
Central	36	7.1%	
Northern	12	2.4%	
Western	366	71.8%	
Eastern	60	11.8%	
Southern	36	7.1%	
Gender			0.047
Male	42	8.2%	
Female	268	91.8%	
Age - Mean Age: 36.68 years Standard Deviation: 9.62 year			0.041
<25 years	156	30.58%	
25-34 years	254	49.80%	
35-44 years	56	10.98%	
45-54 years	26	5.09%	
55 years and above	18	3.52%	
Profession			0.053
Physician	114	22.35%	
Nurses	84	16.47%	
Dietitian	136	46.27%	
Pharmacist	52	10.19%	
Other:	24	4.70%	
Experience			0.029
<5 years	178	34.90%	
5-10 years	180	35.29%	
10-15 years	62	12.15%	
15-20 years	50	9.8%	
>20 years	40	7.8%	
Place of Work			0.033
Hospitals	314	61.56%	
Clinics	66	12.94%	
Rehabilitation centers	98	19.21%	
Other:	32	6.27%	

Awareness and practice

The awareness and practice section focused on the participants' understanding of wound care supplements. A significant proportion encountered pressure ulcers or wounds frequently (36.5%). Awareness levels varied for the use of supplemental vitamin C, arginine, and zinc, with notable percentages of respondents being partially aware or not familiar. Participants demonstrated a diverse understanding of the effectiveness of these supplements, with the majority perceiving them as very effective (52.9%). A substantial portion (42.4%) acknowledged existing guidelines or protocols, while confidence levels in applying these supplements varied. Collaboration and communication among healthcare professionals were generally perceived as good, although there were instances of fair or poor collaboration and communication. The survey questionnaire response rate absolute number (total=510) along with response rate percentage with p-value (p ≤ 0.05) for the knowledge and awareness were tabulated in detail (Table 2).

**Table 2:** Awareness and practice

Survey Questionnaire	Frequency (total=510)	Percentage	P-value (p ≤ 0.05)
How often do you encounter patients with pressure ulcers or wounds in your daily practice?			0.055
Rarely	156	30.6%	
Occasionally	168	32.9%	
Frequently	186	36.5%	
Are you aware of the use of supplemental Vitamin C in the treatment of pressure ulcers and wounds?			0.044
Yes	240	47.1%	
No	132	25.9%	
Partially	138	27.1%	
How familiar are you with the application of Arginine for managing pressure ulcers and wounds?			0.048
Very familiar	168	32.9%	
Somewhat familiar	222	43.5%	
Not familiar at all	120	23.5%	
To what extent do you understand the role of Zinc in wound healing and treating pressure ulcers?			0.039
Very well	138	27.1%	
Moderately well	270	52.9%	
Not well at all	102	20.0%	
In your opinion, how effective are supplemental vitamins C, Arginine and Zinc in promoting wound healing?			0.022
Very effective	270	52.9%	
Moderately effective	204	40.0%	
Slightly effective	24	4.70%	
Not effective at all	12	2.4%	
Are you aware of any existing guidelines or protocols related to the use of supplemental Vitamin C, Arginine, Zinc in pressure ulcer and wound management?			0.045
Yes	116	42.4%	
No	144	28.2%	
Unsure	150	29.4%	
How confident do you feel in applying these supplements in your daily healthcare practice?			0.028
Very confident	186	36.5%	
Moderately confident	216	42.4%	
Slightly confident	72	14.1%	
Not confident at all	36	7.1%	
How would you rate the collaboration between healthcare professionals regarding the use of these supplements?			0.026
Excellent	54	10.6%	
Good	116	42.4%	
Fair	138	27.1%	
Poor	78	15.3%	
No Collaboration	24	4.7%	
Is there effective communication within your healthcare team regarding the benefits and challenges of these supplements in wound care?			0.023
Very effective	54	10.6%	
Moderately effective	216	42.4%	
Slightly effective	120	23.5%	
Not effective at all	36	7.1%	
No Communication	84	16.5%	

Information and training

Regarding information and training, the majority of respondents (64.7%) had received formal training on the application of supplemental vitamin C, arginine, and zinc in wound care. An overwhelming interest in additional training programs was observed (85.9%). A consensus among respondents (60.0%) emphasized the need for increased awareness and education in wound care supplements. Participants relied on various sources of information, with medical journals being the most common (43.5%). Specific topics of interest included advanced wound care techniques, practical applications of Arginine, and the latest research on supplemental Vitamin C. The survey questionnaire response rate absolute number (total=510) along with response rate percentage with p-value (p ≤ 0.05) for the information and training were tabulated in detail (Table 3).

**Table 3:** Information and training

Survey Questionnaires	Frequency (total=510)	Percentage	P-value (p ≤ 0.05)
Have you received formal training on the application of supplemental Vitamin C, Arginine, and Zinc in wound care?			0.035
Yes	230	64.7%	
No	120	23.5%	
In progress	60	11.8%	
Would you be interested in additional training or educational programs related to the use of these supplements in wound management?			0.036
Yes	438	85.9%	
No	6	1.17%	
Maybe	66	12.9%	
Do you believe there is a need for increased awareness and education among healthcare workers regarding the benefits of using supplemental Vitamin C, Arginine, Zinc in wound care?			0.021
Strongly agree	306	60.0%	
Agree	180	35.3%	
Neutral	24	4.7%	
Disagree	0	0.0%	
Strongly disagree	0	0.0%	
What sources do you rely on for information regarding the latest advancements in wound care and treatment?			0.038
Medical journals	210	43.5%	
Conferences/seminars	42	8.2%	
Professional associations	54	10.6%	
Online resources	120	23.5%	
Colleagues/peers	66	12.9%	
Other:	18	3.5%	
What specific topics or areas related to wound care and supplements would you like to learn more about?			0.055
Advanced wound care techniques	120	23.5%	
Latest research on supplemental Vitamin C	60	11.8%	
Practical applications of Arginine in wound healing	102	20.0%	
In-depth understanding of Zinc's role in wound management	30	5.9%	
Interdisciplinary collaboration in wound care	54	10.6%	
Patient education on supplement use	24	4.7%	
Case studies on successful supplement applications	96	18.8%	
Emerging trends in wound care	24	4.7%	
Other:	0	0.0%	

Barriers and challenges

Challenges in incorporating supplements into wound care were identified, including a lack of knowledge, limited resources, time constraints, patient compliance issues, and resistance from colleagues. Barriers to implementing supplemental Vitamin C included limited resources, patient and colleague awareness, and insufficient training. Challenges in understanding the practical applications of Arginine included limited educational resources, unclear guidelines, difficulty accessing relevant information, and limited practical training opportunities. For promoting the use of Zinc supplements, challenges included patient reluctance, a lack of evidence-based information, resistance from colleagues, and limited awareness among healthcare professionals. The survey questionnaire response rate absolute number (total=510) along with response rate percentage with p-value (p ≤ 0.05) for the barriers and challenges were tabulated in detail (Table 4).

**Table 4:** Barriers and challenges

Survey Questionnaires	Frequency (total=510)	Percentage	P-value (p ≤ 0.05)
What challenges do you face in incorporating supplemental Vitamin C, Arginine, Zinc in the treatment of pressure ulcers and wounds?			0.044
Lack of knowledge	144	28.2%	
Limited resources	210	41.2%	
Time constraints	42	8.2%	
Patient compliance	78	15.3%	
Resistance from colleagues	24	4.7%	
Other:	12	2.4%	
What challenges do you perceive in implementing supplemental Vitamin C in wound care?			0.037
Limited availability of resources	180	35.3%	
Lack of awareness among patients	126	24.7%	
Resistance from patients or colleagues	42	8.2%	
Insufficient training	156	30.6%	
Other:	6	1.17%	
Are there any barriers you face in understanding the practical applications of Arginine in wound management?			0.043
Limited educational resources	138	27.1%	
Lack of clear guidelines	168	32.9%	
Difficulty in accessing relevant information	48	9.4%	
Limited practical training opportunities	150	29.4%	
Other:	6	1.17%	
Do you encounter challenges in promoting the use of Zinc supplements for wound healing?			0.044
Patient reluctance	90	17.6%	
Lack of evidence-based information	126	24.7%	
Resistance from colleagues	30	5.9%	
Limited awareness among healthcare professionals	246	48.2%	
Other:	18	3.5%	

Importance of nutritional supplements

Participants were asked to rate the importance of nutritional supplements on a scale of 1 to 10, with 10 being the highest. The results indicated a significant acknowledgment of the importance of nutritional supplements in the management of pressure ulcers and wounds, with the majority rating it 8 and above. The survey questionnaire response rate absolute number (total=510) along with response rate percentage with p-value (p ≤ 0.05) for the importance of nutritional supplements were tabulated in detail (Table 5).

**Table 5:** Importance of nutritional supplements

Survey Questionnaires	Frequency (total=510)	Percentage	P-value (p ≤ 0.05)
On a scale of 1 to 10, how would you rate the importance of nutritional supplements, including Vitamin C, Arginine, and Zinc, in the management of wounds?			0.048
1	18	3.5%	
2	0	0.0%	
3	12	2.4%	
4	12	2.4%	
5	18	3.5%	
6	24	4.7%	
7	54	10.6%	
8	102	20.0%	
9	60	11.8%	
10	210	41.2%	

The survey responses provided valuable insight into the knowledge, awareness, practice, training needs, and challenges faced by healthcare workers regarding the use of supplemental vitamin C, arginine, and zinc in wound care. The findings suggest a positive inclination toward additional education and training programs to enhance understanding and application in daily healthcare practices. Addressing challenges and promoting collaborative efforts could contribute to improved wound care practices among healthcare professionals.

## Discussion

This comprehensive survey study aimed to understand the knowledge, awareness, practices training needs, and challenges faced by healthcare workers in Saudi Arabia regarding the application of supplemental vitamin C, arginine, and zinc in managing wounds. The study also explored the importance of nutritional supplements in wound care.

The awareness and practice delved into participants' grasp of wound care supplements, revealing that a substantial proportion encountered pressure ulcers or wounds frequently (36.5%). Awareness varied for supplemental vitamin C, arginine, and zinc, with notable percentages partially aware or unfamiliar. Participants demonstrated diverse views on the effectiveness of these supplements, with the majority perceiving them as highly effective (52.9%). While 42.4% acknowledged existing guidelines, confidence in application varied. Collaboration among healthcare professionals was generally viewed positively, though instances of fair or poor collaboration were noted. The detailed tabulation of survey responses (total=510) and percentages with a p-value (p ≤ 0.05) for knowledge and awareness was presented. Regarding information and training, 64.7% had formal training on supplement application, and 85.9% expressed interest in additional programs. A consensus (60.0%) emphasized the need for increased awareness. Survey responses (total=510) and percentages with p-value (p ≤ 0.05) for information and training were thoroughly detailed. Identified challenges in supplement integration included lack of knowledge, limited resources, and patient compliance issues. Barriers specific to each supplement were outlined. Participants affirmed the importance of nutritional supplements in managing pressure ulcers and wounds, with the majority rating it 8 and above. The detailed tabulation of survey responses (total=510) and percentages with p-value (p ≤ 0.05) for the importance of nutritional supplements was also provided.

The demographic data indicated a strong representation from the Western region and a predominance of female respondents. The majority fell within the 25-34 age group, and dietitians constituted the largest professional group. Hospitals were the primary workplace, and a significant proportion of respondents had less than 10 years of experience. These demographics provide a snapshot of the diverse backgrounds and experiences of healthcare professionals participating in the survey.

The survey revealed varying levels of awareness and understanding among healthcare workers regarding the use of supplemental vitamin C, arginine, and zinc. While a substantial proportion perceived these supplements as very effective, some respondents were only partially aware of them or not familiar. The existence of guidelines or protocols was acknowledged by a considerable percentage. Confidence levels in using these supplements varied, indicating potential areas for targeted education and training. Collaboration and communication among healthcare professionals were generally viewed positively, although there were instances of fair or poor collaboration and communication.

The majority of respondents had received formal training on the application of supplements, and there was a notable interest in additional training programs. A consensus among respondents emphasized the need for increased awareness and education in wound care supplements. Medical journals were the primary source of information, indicating a reliance on scholarly literature. Specific topics of interest included advanced wound care techniques, practical applications of Arginine, and the latest research on supplemental Vitamin C.

Arginine, identified as a crucial amino acid, serves as a building block for cell growth, collagen deposition, and lymphocyte function [21]. Its significance lies in being the biological precursor for nitric oxide (NO), a molecule known for its substantial impact on wound healing. Studies have illustrated that nitric oxide metabolites positively regulate wound repair, with reduced levels observed in the wound environments of individuals with diabetic ulcers [22]. Arginine also acts as a precursor for proline, an essential component for collagen synthesis [23].

Zinc, another essential element, is required for numerous enzymes involved in wound healing. Its role extends to tissue repair, growth, antioxidant function, and immune system support [24]. Zinc is crucial for the synthesis of collagen, DNA, RNA, and proteins, playing a particularly vital role in cellular proliferation [19]. Instances of chronic wounds with excessive drainage reduced dietary intake, or excessive gastrointestinal losses have been associated with zinc deficiency [25].

Vitamin C, renowned for its contributions to collagen synthesis, immune system modulation, and antioxidant capabilities, plays a significant role in wound healing [26]. Deficiency in vitamin C is linked to reduced collagen production and hindered wound healing [27]. Research has indicated that the benefits of vitamin C supplementation are enhanced when administered in conjunction with zinc and arginine [28].

The challenges in incorporating supplements into wound care were identified, including a lack of knowledge, limited resources, time constraints, patient compliance issues, and resistance from colleagues. Specific barriers for each supplement were highlighted, such as limited availability of resources for Vitamin C, patient and colleague awareness for Arginine, and patient reluctance for zinc [29]. These challenges underscore the importance of addressing both knowledge gaps and practical obstacles to ensure the effective integration of supplements into wound care practices. Pressure ulcers, acknowledged as a significant healthcare challenge, pose complications for patients and exert substantial economic burdens on healthcare systems [30]. As the Kingdom of Saudi Arabia undergoes advancements in its healthcare infrastructure, it becomes imperative to evaluate the depth of healthcare workers' understanding of innovative approaches to wound care [29]. The incorporation of supplemental nutrients, renowned for their roles in collagen synthesis, immune function, and tissue repair, emerges as a promising avenue for improving patient outcomes [30].

The participants recognized the significant importance of nutritional supplements, with the majority rating it 8 and above on a scale of 1 to 10. This high rating indicates a strong acknowledgment of the role these supplements play in the management of pressure ulcers and wounds. This research endeavors to evaluate the knowledge base of healthcare workers in Saudi Arabia concerning the use of supplemental vitamin C, arginine, and zinc in wound management [12]. By comprehending the awareness levels among healthcare workers, the study aims to pinpoint potential knowledge gaps that might impede the optimal integration of these nutritional interventions into clinical practice [13]. The Kingdom's unique healthcare landscape, characterized by a diverse healthcare workforce and a growing emphasis on research and evidence-based practice, provides a compelling context for this investigation. By gauging healthcare workers' familiarity with these supplements and their perceived effectiveness, the study seeks to contribute valuable insights that can inform targeted educational initiatives, and policy considerations, and, ultimately, enhance the quality of wound care in Saudi Arabian healthcare settings [14].

The survey results provide valuable insights into the current landscape of knowledge, awareness, and challenges faced by healthcare professionals in Saudi Arabia concerning nutritional supplements in wound care. The findings suggest opportunities for targeted education and training programs, collaborative initiatives, and addressing specific challenges to enhance the effective utilization of these supplements in clinical practice. Continued research and efforts toward improving awareness and knowledge dissemination can contribute to advancements in wound care practices in the healthcare community.

Strengths and weakness

The study boasts several strengths, including the comprehensive cross-sectional research design that provides a thorough snapshot of healthcare professionals' knowledge and awareness levels regarding supplemental vitamin C, arginine, and zinc in wound management. The robust questionnaire covers diverse aspects, from demographic details to psychosocial factors, ensuring a holistic understanding of the subject. Translation and cultural adaptation enhance the questionnaire's accessibility for the Saudi Arabian target population. The validation process, involving nutrition and statistics experts with a pilot study, ensured reliable survey instruments. Determining an adequate sample size adds statistical rigor, while meticulous ethical considerations safeguard participant rights and data integrity. Thorough data analysis contributes to a robust exploration of socio-demographic composition and knowledge levels. Despite these strengths, the study exhibits weaknesses, including regional imbalance, gender disparity, professional representation, potential biases in self-reported data, limited exploration of influencing factors, and the cross-sectional nature, which need addressing for enhanced generalizability and a more nuanced understanding. Future research efforts should aim to mitigate these weaknesses for improved overall robustness and applicability in informing healthcare practices in Saudi Arabia.

## Conclusions

This comprehensive cross-sectional survey study sheds light on the awareness and practices of healthcare workers in Saudi Arabia concerning the use of supplemental vitamin C, arginine, and zinc in managing wounds. The study's robust cross-sectional design encompasses a diverse range of socio-demographic variables. The questionnaire's meticulous development, translation, and validation ensured its relevance and reliability for the target population. The findings reveal both strengths and weaknesses in healthcare worker's understanding and use of nutritional supplements. There was a positive inclination toward the perceived effectiveness of these supplements, challenges such as limited resources and knowledge gaps need addressing. The study underscores the importance of additional training programs and collaborative initiatives to enhance wound care practices. It contributes valuable insights for healthcare workers, policymakers, and educators, aiming to advance wound care practices and improve patient outcomes in the unique healthcare landscape of Saudi Arabia. Future research and interventions should focus on addressing identified challenges and promoting collaborative efforts among healthcare professionals in the region.
